# New Validated Staging System for Light Chain (AL) Amyloidosis With Stage IIIC Defining Ultra-Poor Risk: AL International Staging System

**DOI:** 10.1200/JCO-25-02558

**Published:** 2025-12-07

**Authors:** Jahanzaib Khwaja, Amy A. Kirkwood, Paolo Milani, Binoy Yohannan, Foteini Theodorakakou, Flores Weverling, Valeria Di Simone, Sriram Ravichandran, Shaji Kumar, Ioannis Petropoulos, Roberta Mussinelli, Oliver Cohen, Marish I.F.J. Oerlemans, Eli Muchtar, Kimon Stamatelopoulos, Helen J. Lachmann, Julian D. Gillmore, Alexandros Briasoulis, Morie Gertz, Carol Whelan, Lucia Venneri, Marianna Fontana, Shameem Mahmood, Rahel Schwotzer, Monique C. Minnema, Angela Dispenzieri, Giovanni Palladini, Efstathios Kastritis, Ashutosh Wechalekar

**Affiliations:** ^1^ National Amyloidosis Centre, Royal Free London, UCL, London, United Kingdom; ^2^ University College London Hospitals, London, United Kingdom; ^3^ CRUK and UCL Cancer Trials Centre, UCL Cancer Institute, UCL, London, United Kingdom; ^4^ Department of Molecular Medicine, University of Pavia, Pavia, Italy; ^5^ Amyloidosis Research and Treatment Center, Fondazione IRCCS Policlinico San Matteo, Pavia, Italy; ^6^ Mayo Clinic, Rochester, MN; ^7^ Alexandra General Hospital, National and Kapodistrian University of Athens, Athens, Greece; ^8^ University Medical Center Utrecht, University Utrecht, Utrecht, the Netherlands; ^9^ Division of Cardiology, Fondazione IRCCS Policlinico San Matteo, Pavia, Italy; ^10^ University Hospital Zurich, Zurich, Switzerland

## Abstract

**PURPOSE:**

Outcomes in systemic light chain (AL) amyloidosis have improved with modern therapy limiting utility of existing risk stratification models. We validate a new staging system, incorporating longitudinal strain (LS) to the biomarker-based (NT-proBNP and Troponin-T) staging system in the contemporary treatment era (2015-2024).

**METHODS:**

AL International Staging System (AL-ISS) was derived from a cohort of patients with AL amyloidosis from the UK National Amyloidosis Centre (2015-2019). The model was validated in patient cohorts from Europe (Greece, Italy, the Netherlands, and Switzerland), the United States (2015-2024), and the United Kingdom (2020-2024).

**RESULTS:**

In total, 2,493 patients were included (derivation, n = 573; validation n = 1,920). In a multivariable model for the derivation cohort, LS ≥ –9% and cardiac biomarkers at previously validated thresholds (NT-proBNP 332 ng/L and 8,500 ng/L and high-sensitivity troponin T ≥ 50 ng/L) were independent poor prognostic factors stratifying patients into stages I, II, IIIA, IIIB, and IIIC. In the validation cohort, the patient stages were stage I: 317 (17%), II: 782 (41%), IIIA: 551 (29%), IIIB: 174 (9%), and IIIC: 96 (5%), respectively (first-line daratumumab treated: 826; 43%). With a median follow-up of 34 months, median overall survival (OS) was not reached (NR); estimated 1-year, 2-year, and 3-year OS was 82%, 74%, and 70% respectively. The median survival for stages I to II, IIIA, IIIB, and IIIC were NR, 67, 26, and 7 months (1-year OS IIIC 53% *v* 68% for IIIB in the daratumumab-treated patients), respectively (*P* < .001). External validation exhibited good predictive performance: 12-month calibration slope was 1.09, Harrell C 0.69, Royston D 1.19, and R^2^_D_ 0.25. Stage IIIC independently discriminated the poorest outcome across all cohorts.

**CONCLUSION:**

This defines and validates a new staging system from systemic AL amyloidosis with robust identification of an ultra-poor risk stage (IIIC) in contemporarily treated patients.

## INTRODUCTION

Systemic light chain (AL) amyloidosis is an incurable disorder caused by misfolded light chain protein fibrils from an underlying plasma cell clone, causing organ dysfunction. Most patients present with cardiac involvement, and survival is determined by the extent of cardiac involvement as well as response to antiplasma cell therapy.^1,2^

**CONTEXT**

**Key Objective**
To externally validate a new staging system for systemic light chain (AL) amyloidosis (AL International Staging system [AL-ISS]) in the modern treatment era.
**Knowledge Generated**
AL-ISS was derived from 573 patients in the United Kingdom and externally validated in an independent cohort of 1,920 patients in Europe, the United States, and the United Kingdom. Stage I, II, and IIIA were defined as previously described in the European modification of Mayo 2004 staging. Stage IIIB and IIIC were newly defined as IIIB: longitudinal strain (LS) < –9%, NT-proBNP ≥ 8,500 ng/L, high-sensitivity troponin T (hs-TnT) ≥ 50 ng/L; IIIC: LS ≥ –9%, NT-proBNP ≥ 8,500 ng/L, hs-TnT ≥ 50 ng/L with a median survival of 26 and 7 months, respectively
**Relevance *(S. Lentzsch)***
The AL-ISS enables clinicians to identify AL amyloidosis patients at the highest risk with far greater precision, guiding earlier intervention and closer cardiac management; for patients who meet stage IIIC criteria, the staging supports proactive decisions, such as expedited referral for cardiac transplant evaluation. Its broad international validation positions AL-ISS as the new standard framework for both routine care and future trial development.*
*Relevance section written by *JCO* Associate Editor Suzanne Lentzsch, MD, PhD.

Risk stratification of newly diagnosed patients is critical, determining prognosis and treatment planning in addition to appropriate selection for clinical trials. The two most widely used prognostic staging systems are the European modification of Mayo 2004 (stage I to IIIB)^3,4^ and Mayo 2012 (stage I to IV).^5^ These are based solely on blood cardiac biomarkers (NT-proBNP and cardiac troponin) for modified Mayo 2004 and the addition of light-chain burden (difference in the involved and uninvolved free light chain [dFLC]) for Mayo 2012. Patients in these original studies were treated with now historic, heterogeneous regimens. Their modern predictive ability has been questioned.^6-8^ Although both are useful in the bortezomib-treated era, the European modification was more discriminatory of early outcome than Mayo 2012.^6^ Lately, daratumumab-bortezomib–based first-line quadruplets have dramatically improved hematological responses translating to overall survival (OS) benefit and the first drug approval in AL amyloidosis.^9^ During this time, survival of the traditionally poorest group, stage IIIb, has improved from <6 months to well over 12 months with medians not reached (NR) in other stages.^10-12^ In short, current models fail to identify patients at the highest risk, underscoring the need for a new staging system to more effectively stratify patients in the contemporary era.

Echocardiography is a standard diagnostic tool recommended in all patients. Longitudinal strain (LS) measures the percentage change in longitudinal myocardial shortening, and has emerged as a sensitive marker of left ventricular (LV) dysfunction. LS is expressed as a negative value. A more negative number indicates a greater degree of shortening (normal is –18% or more negative). With worsening cardiac amyloid deposition, there is less longitudinal contractility (reduced vertical motion of the cardiac apex in systole) and strain moves closer to zero (ie, more negative the value—better, less negative the value—worse). We have reported the independent predictive value of poor LS on outcomes in AL amyloidosis.^13^ We previously proposed a new staging model incorporating LS into the biomarker-based European model, defining the ultra-poor risk group, stage IIIC in a single center study in the bortezomib-era.^7^ The new staging system is defined by the combination of NT-proBNP ≥ 8,500 ng/L, high-sensitivity troponin T (hs-TnT) ≥ 50 ng/L and LS ≥ –9%.^7^

In this study, we aimed to externally validate this model in a large cohort of patients from the United Kingdom, Europe, and the United States treated in the modern era. We termed this new staging system the AL International Staging System (AL-ISS).

## METHODS

### Derivation Cohort

AL-ISS was developed from newly diagnosed patients with systemic AL from the UK National Amyloidosis Centre (NAC) between 2015 and 2019. Histopathologic diagnosis was confirmed on polarizing light microscopy with characteristic apple-green birefringence on Congo red-stained tissue and fibril typing by immunohistochemistry/mass spectrometry and hereditary amyloidosis ruled out by appropriate genetic screening when clinically indicated. Standardized assessments including standard biomarker measurement, clonal parameters, and imaging were performed, reported as per International Society of Amyloidosis consensus criteria.^14^ All had LS measurement as per previously published guidance^15^ (Data Supplement [Supplementary Methods], online only).

The primary end point was OS, defined as time from diagnosis to death with censoring at last follow-up. OS was analyzed by Kaplan-Meier curves and compared using Cox regression and log-rank test. Prognostic association between explanatory variables and OS was assessed using univariable and multivariable models in the derivation data set. Optimal LS threshold for high risk was determined by time-dependent receiver operating characteristic (ROC) curves among European IIIb and selected alongside traditional biomarkers to propose the AL-ISS as previously reported.^7^ Collinearity of variables was assessed by correlation matrix of estimated coefficients and variance inflation factor (VIF). Outcomes by AL-ISS were reported and exploratory subanalyses conducted comparing outcomes across 2015-2019 and 2020-2024, chronological tertiles, first-line daratumumab-containing and noncontaining regimens and light-chain isotype.

### External Validation

External validation was derived from newly diagnosed patients from Europe (Greece, Italy, the Netherlands, and Switzerland), the United States (Mayo Clinic) both between 2015 and 2024, and the NAC between 2020 and 2024. Written consent was obtained in all, and ethical approval was obtained. Characteristics between groups were compared using χ^2^ or Fisher exact tests (categorical variables) or Wilcoxon Mann-Whitney/Kruskal-Wallis tests (continuous variables) for European/NAC data sets. Data from the United States were analyzed in a federated analysis approach where Mayo institute conducted its own analysis on identifiable data for baseline characteristics, univariable and multivariable analysis using the methods and parameters as described for European/NAC patients. OS by AL-ISS and model diagnostics were performed on pooled data from all centers.

### Model Evaluation

Model performance was evaluated in the validation cohort by overall fit (Royston R^2^_D_) measuring the proportion of the total variance of outcome explained by the model^16^ and calibration plot^17^ comparing observed and predicted survival, reporting the calibration slope (ideal value of 1 with complete agreement) and calibration-in-the-large (ideal value of 0). Discrimination was assessed by Harrell concordance (C) index and Royston D statistic (log hazard ratio comparing two equally sized groups). Higher values (range, 0-1) indicate better discrimination. Sensitivity, specificity, and diagnostic odds ratio (DOR; sensitivity × specificity/[1 – sensitivity] × [1 – specificity]) at 1, 2, and 3 years were reported. Statistical analyses were conducted using Stata v19 (StataCorp, College Station, TX).

## RESULTS

A total of 2,493 patients were analyzed from 2015 to 2024: 573 derivation and 1,920 external validation (Europe and the United States: Greece n = 163, Italy n = 118, the Netherlands n = 59, Switzerland n = 34, Mayo n = 458; NAC, n = 1,088; supplemental results). The NAC derivation cohort was diagnosed between 2015 and 2019, while the validation cohort was diagnosed between 2020 and 2024. Baseline characteristics are outlined in Table 1.

**TABLE 1.** Baseline Characteristics of Derivation and Validation Cohorts

Characteristic	Derivation	External Validation		
	NAC, 2015-2019 (n = 573)	NAC, 2020-2024 (n = 1,088)	European, 2015-2024 (n = 374)	Mayo, 2015-2024 (n = 458)
Age, years, median (IQR)	67 (60-73)	67 (60-74)	66 (60-73)	66 (59-72)
Male, No. (%)	348 (61)	633 (58)	222 (59)	291 (64)
Female, No. (%)	225 (39)	455 (42)	152 (41)	167 (36)
AL isotype, No. (%)^a^				
λ type	450 (79)	803 (74)	299 (80)	347 (76)
κ type	123 (21)	285 (26)	74 (20)	111 (24)
Bone marrow plasma cell infiltrate, %, median (IQR)^b^	10 (6-20)	13 (10-20)	13 (8-20)	10 (6-20)
dFLC, mg/L, median (IQR)^a^	194 (82-509)	169 (75-409)	229 (96-505)	231 (93-554)
Organ involvement, No. (range)	2 (1-5)	1 (1-5)	2 (1-6)	2 (1-5)
Organ involvement, No. (%)^a^				
Cardiac	404 (71)	682 (64)	322 (87)	308 (67)
Renal	402 (70)	659 (61)	210 (57)	247 (54)
Liver	57 (10)	105 (10)	61 (17)	63 (14)
NT-proBNP, ng/L, median (IQR)^a^	2,317 (729-5,806)	1,911 (533-5,174)	2,734 (963-7,173)	1,779 (448-4,610)
Hs-Tn T, ng/L, median (IQR)^a^	57 (28-118)	44 (23-81)	48 (26-78)	44 (24-95)
LS, %, median (IQR)	–14.9 (–10.7 to −19.3)	–14.9 (–11 to −19)	–14.0 (–11 to −18)	–14.6 (–10 to −18)
Supine systolic blood pressure, mm Hg, median (IQR)^a^	118 (107-133)	121 (109-136)	117 (105-132)	113 (102-125)
Creatinine, µmmol, median (IQR)	97 (77-143)	96 (74-140)	94 (72-122)	102.1 (80-144)
Proteinuria, g/24 h, median (IQR)^a^	2.8 (0.5-6.5)	1.6 (0.3-5.7)	1.4 (0.3-5.9)	1.6 (0.2-5.3)
Alkaline phosphatase, U/L, median (IQR)^a^	92 (70-130)	96 (76-125)	84 (66-116)	89 (71-124)
Albumin, g/L, median (IQR)	35 (27-41)	36 (28-41)	35 (29-40)	32 (27-37)
AL-ISS stage, No. (%)^a^				
I	92 (16)	188 (17)	36 (10)	93 (20)
II	169 (29)	426 (39)	191 (51)	165 (36)
IIIA	225 (39)	325 (30)	79 (21)	147 (32)
IIIB	52 (9)	100 (9)	41 (11)	33 (7)
IIIC	35 (6)	49 (5)	27 (7)	20 (4)
First-line daratumumab-based treatment, No. (%)^a^	0 (0)	431 (40)	161 (43)	234 (51)
Year of diagnosis, No. (%)				
2015-2019	573 (100)	—	95 (25)	179 (39)
2020-2024	—	1,088 (100)	279 (75)	279(61)

Abbreviations: AL, light chain, AL-ISS, AL International Staging system; dFLC, difference in the involved and uninvolved free light chain; LS, longitudinal strain; NA, not available.

^a^ *P* < .05 across European, NAC derivation, and NAC validation cohorts.

^b^ Bone marrow infiltrate available in 699 (258/573 NAC, 120/1088 NAC validation, 321/374 European).

### Derivation Cohort

The median age at diagnosis was 67 years (IQR, 60-73), 79% had lambda AL-type with a median plasma cell infiltrate of 10% (IQR, 6-20); 71% had cardiac and 70% renal involvement. The median dFLC was 194 mg/L (IQR, 82-509), NT-proBNP 2317 ng/L (IQR, 729-5,806), hs-TnT 57 ng/L (IQR, 28-118), and LS –14.9% (IQR, –10.7 to –19.3). The median supine systolic blood pressure was 118 mm Hg (IQR, 107-133), LV septal thickness was 14 mm (IQR, 12-16), ejection fraction 59% (IQR, 52-63), proteinuria 2.8 g/24 hours (IQR, 0.5-6.5), and alkaline phosphatase (ALP) 92 U/L (IQR, 70-130).

### AL-ISS Model

On univariable analysis, European staging biomarkers, dFLC ≥180 mg/L (hazard ratio [HR], 1.47 [95% CI, 1.12 to 1.91]), LS ≥ –9% (HR, 2.98 [95% CI, 2.21 to 4.01]; *P* < .001), and supine systolic blood pressure <100 mm Hg (HR, 1.60 [95% CI, 1.11 to 2.31]; *P* = .01) were significant predictors of OS. The optimal threshold of LS ≥ –9% was selected based on previous reports^13^ and time-dependent ROC analysis for European IIIb (Data Supplement, Tables S2 and S3). On multivariable analysis including dFLC ≥180 mg/L, LS ≥ –9% and European modified staging, only dFLC ≥ 180 mg/L was not independently predictive of OS (Table 2). The addition of supine systolic blood pressure < 100 mm Hg, previously identified as a poor prognostic factor, was not independently predictive (*P* = .64). There was no collinearity of covariates (NT-proBNP ≥ 8,500 ng/L, hs-TnT ≥ 50 ng/L, LS ≥ –9%): pairwise correlations were low (|ρ| < .3) and VIF < 5 (range, 1.07-1.19, mean VIF, 1.15). LS ≥ –9% was also tested in all stages (Data Supplement, Fig S1), but remained most predictive for poor outcomes in substratifying previously-defined stage IIIb.

**TABLE 2.** Predictors of Overall Survival by dFLC ≥ 180 mg/L, LS ≥ –9% With European Modified Staging System and AL-ISS

Characteristic	Derivation				Validation (European/NAC)				Validation (Mayo 2015-2024)			
	Univariable		Multivariable		Univariable		Multivariable		Univariable		Multivariable	
	HR (95% CI)	*P*	HR (95% CI)	*P*	HR (95% CI)	*P*	HR (95% CI)	*P*	HR (95% CI)	*P*	HR (95% CI)	*P*
Model 1: dfLC > 180 mg/L and LS > –9% with European modified staging system												
dFLC ≥180 mg/L	1.46 (1.12 to 1.91)	.005	1.10 (0.83 to 1.44)	.51	1.30 (1.07 to 1.57)	.009	1.01 (0.82 to 1.25)	.91	1.37 (0.99 to 1.92)	.05	1.11 (0.73 to 1.70)	.62
LS ≥ –9%	2.98 (2.21 to 4.01)	<.001	1.94 (1.41 to 2.66)	<.001	2.47 (1.94 to 3.16)	<.001	1.55 (1.19 to 2.02)	.001	3.42 (2.30 to 5.01)	<.001	2.19 (1.45 to 3.29)	<.001
Stage I												
II	2.28 (1.24 to 4.21)	<.001^a^	2.12 (1.14 to 3.93)	<.001	2.01 (1.32 to 3.07)	<.001^a^	1.86 (1.19 to 2.91)	<.001	2.18(1.16 to 4.48)	<.001^a^	2.09 (0.92 to 5.62)	<.001
IIIa	4.66 (2.62 to 8.28)		3.96 (2.20 to 7.11)		3.51 (2.30 to 5.35)		2.96 (1.88 to 4.67)		4.09(2.22 to 8.27)		3.23 (1.43 to 8.67)	
IIIb	8.96 (4.90 to 16.38)		6.95 (3.73 to 12.93)		7.16 (4.68 to 10.96)		5.92 (3.72 to 9.41)		15.41(8.20 to 31.56)		10.67 (4.67 to 28.89)	
Model 2: dFLC >180 mg/L with AL-ISS biomarkers												
dFLC ≥180 mg/L	1.46 (1.12 to 1.91)	.005	1.10 (0.83 to 1.45)	.51	1.30 (1.07 to 1.57)	.009	0.98 (0.80 to 1.20)	.84	1.37 (0.99- 1.92)	.05	0.88 (0.62 to 1.26)	.51
AL-ISS stage I												
II	2.29 (1.24 to 4.22)	<.001^a^	2.24 (1.21 to 4.45)	<.001	2.01 (1.32 to 3.07)	<.001^a^	2.02 (1.32 to 3.08)	<.001	2.17(1.15 to 4.49)	<.001^a^	2.18 (1.16 to 4.48)	<.001
IIIA	4.67 (2.63 to 8.31)		4.53 (2.52 to 8.11)		3.51 (2.30 to 3.35)		3.54 (2.32 to 5.40)		4.06(2.20 to 8.21)		4.17 (2.25 to 8.46)	
IIIB	6.26 (3.25 to 12.05)		6.17 (3.20 to 11.90)		5.67 (3.60 to 8.91)		5.69 (3.60 to 8.99)		12.03 (6.04 to 25.57)		12.52 (6.22 to 26.85)	
IIIC	16.07 (8.32 to 31.04)		15.28 (7.78 to 30.01)		11.13 (6.93 to 17.87)		11.00 (6.78 to 17.85)		23.47 (11.15 to 51.66)		24.97 (11.60 to 56.19)	

Abbreviations: AL-ISS, AL International Staging system; dFLC, difference in the involved and uninvolved free light chain; LS, longitudinal strain.

^a^ Log-rank test for trend.

LS ≥ –9% was added to IIIb biomarkers to define the ultra-poor risk group, stage IIIC. We therefore defined AL-ISS as stage I, II, and IIIA as previously described in the European modification of Mayo 2004 staging. Stage IIIB and IIIC were defined as follows IIIB: LS < –9%, NT-proBNP ≥8,500 ng/L, and hs-TnT ≥50 ng/L and IIIC: LS ≥ –9%, NT-proBNP ≥8,500 ng/L, and hs-TnT ≥50 ng/L.

Applying the AL-ISS, in the derivation cohort the patients staged as stage I: 92 (16%), II: 169 (29%), IIIA: 225 (39%), IIIB: 52 (9%), and IIIC: 35 (6%). The median LS was –20.9% (IQR, –22.4 to –18.2), –16% (IQR, –19 to –16), –12.8% (IQR, –17.0 to –10.1), –12.3% (IQR, –15 to –10.8), –6.8% (IQR, –7.9 to –5.0), across respective stages. The median LS for NT-proBNP ≥8,500 ng/L *v* <8,500 ng/L and hs-TnT ≥50 ng/L *v* <50 ng/L were –10.4% (IQR, –13.3 to –7.2), –16% (IQR, –20 to –11.2), –11.9 (IQR, –16.1 to –9.2) and –21.3 (IQR, –21.3 to –14.3), respectively. The overall median follow-up was 63 months (IQR, 53-74), with estimated median, 1-year, 2-year, and 3-year OS NR, 76% (95% CI, 72 to 79), 67% (95% CI, 63 to 71), and 61% (95% CI, 57 to 65), respectively.

### Validation Cohort

**FIG 1.**
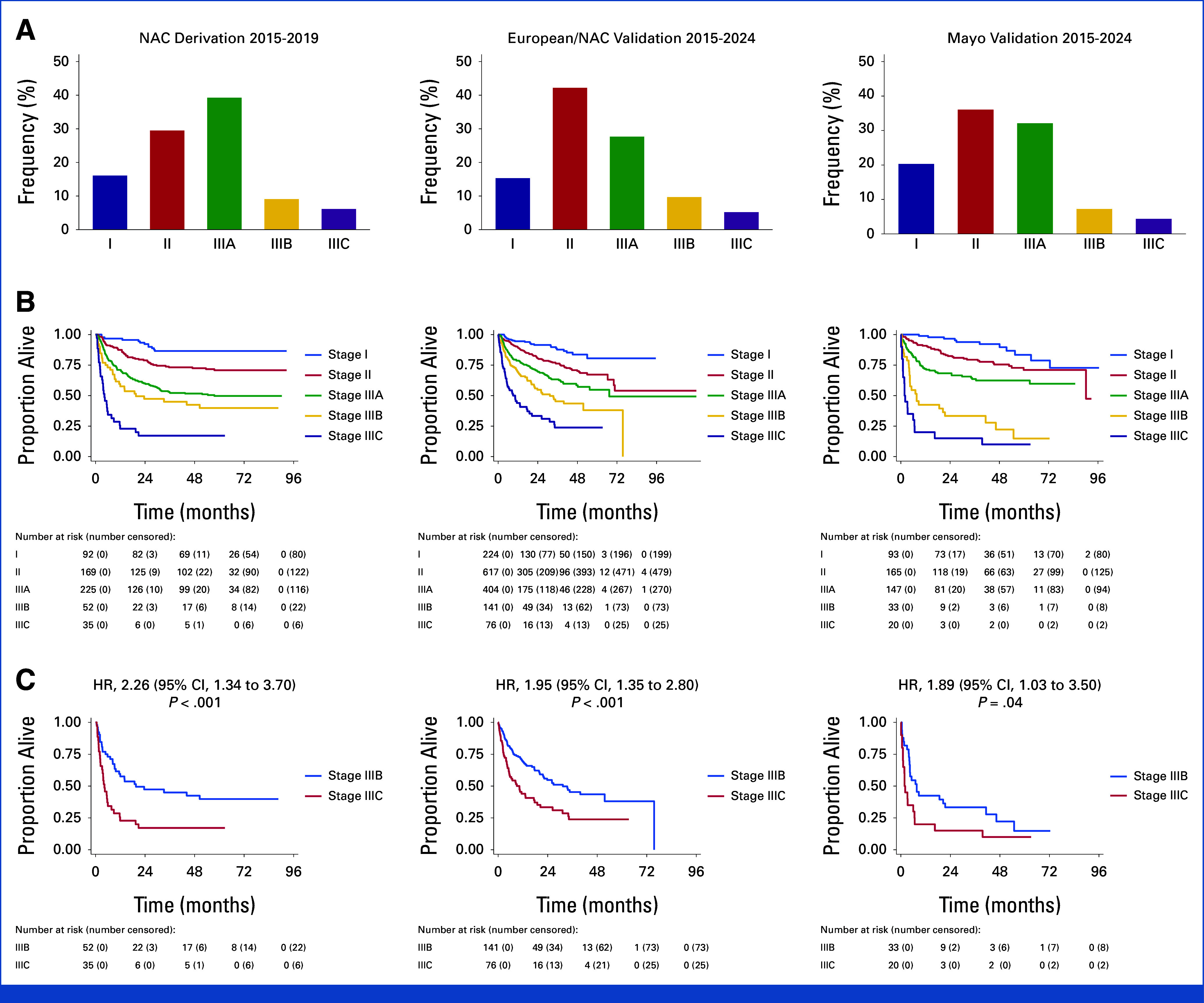
Overall survival by AL-ISS. (A) Proportion by AL-ISS, (B) outcome by AL-ISS, and (C) stage IIIB versus IIIC. AL-ISS, AL International Staging System; HR, hazard ratio; NAC, National Amyloidosis Centre.

Patient characteristics are shown in Table 1. In the European cohort compared with NAC, there was a significantly higher proportion of lambda AL isotype (80% *v* 74%-79%, *P* = .02), cardiac involvement (87% *v* 64%-71%, *P* < .001), higher median cardiac biomarkers, less frequent renal involvement (57% *v* 61%-70%, *P* < .001), lower proteinuria (1.4 *v* 1.6-2.8 g/L, *P* = .003), greater liver involvement (17% *v* 10%, *P* < .001), and lower ALP (84 *v* 92-96 U/L, *P* = .001). The European cohort had a greater proportion of cardiac involvement compared with NAC and Mayo validation groups (87% *v* 64%-67%, *P* < .001). Patient stages were stage I: 317 (17%), II: 782 (41%), IIIA: 551 (29%), IIIB: 174 (9%), and IIIC: 96 (5%), respectively (Fig 1A). Stage IIIA predominated in the derivation cohort, whereas stage II predominated in the validation cohort. A total of 826 patients (43%) were treated with first-line daratumumab-based regimens.

In the validation cohort, at a median follow-up of 34 months (IQR, 20-50), estimated median, 1-year, 2-year, and 3-year OS were NR, 82% (95% CI, 80 to 84), 74% (95% CI, 72 to 76), and 70% (95% CI, 67 to 72), respectively (Fig 1). The median survival for stages I to II, IIIA, IIIB, and IIIC were NR, 67 months (95% CI, 48 to NR), 26 months (95% CI, 19 to 41), and 7 months (95% CI, 4 to 11), respectively (*P* < .001). Hazard ratios for stage II, IIIA, IIIB, and IIIC were 2.17 (95% CI, 1.52 to 3.11), 3.73 (95% CI, 2.61 to 5.34), 6.99 (95% CI, 4.75 to 10.27), and 13.70 (95% CI, 9.14 to 20.54), respectively (*P* < .001; Table 3).

**TABLE 3.** Survival Outcomes by AL-ISS

Outcome	Derivation	External Validation			
	NAC, 2015-2019 (n = 573)	Total Validation (n = 1,920)	NAC, 2020-2024 (n = 1,088)	European, 2015-2024 (n = 374)	Mayo, 2015-2024 (n = 458)
Follow-up, months, median (IQR)	63 (53-74)	34 (20-50)	28 (16-43)	41 (24-55)	46 (31-64)
OS by stage, months, median (95% CI)					
I	NR	NR	NR	NR	NR
II	NR	NR (70 to NR)	NR	NR	90 (90 to NR)
IIIA	58 (30 to NR)	67 (48 to NR)	NR	NR	NR
IIIB	24 (9 to NR)	26 (19 to 41)	34 (21 to NR)	26 (11 to NR)	8 (4 to 22)
IIIC	4 (2 to 6)	7 (4 to 11)	13 (5 to 31)	5 (3 to 18)	2 (1 to 7)
1-year OS (95% CI)					
I	97 (91 to 99)	95 (91 to 97)	94 (89 to 97)	97 (82 to 100)	99 (92 to 100)
II	87 (81 to 92)	89 (97 to 92)	90 (87 to 93)	87 (81 to 91)	91 (85 to 94)
IIIA	71 (65 to 76)	78 (74 to 82)	78 (73 to 83)	79 (68 to 87)	72 (64 to 78)
IIIB	59 (44 to 71)	64 (56 to 70)	72 (62 to 80)	61 (44 to 74)	42 (26 to 58)
IIIC	23 (11 to 38)	39 (29 to 49)	50 (35 to 64)	33 (17 to 51)	20 (6 to 39)
2-year OS (95% CI)					
I	92 (85 to 96)	92 (87 to 95)	90 (85 to 94)	97 (82 to 100)	97 (90 to 99)
II	78 (71 to 84)	81 (77 to 84)	82 (78 to 86)	77 (70 to 83)	82 (76 to 88)
IIIA	60 (53 to 66)	69 (64 to 74)	68 (62 to 73)	72 (60 to 81)	68 (60 to 75)
IIIB	48 (34 to 61)	50 (42 to 58)	54 (43 to 65)	52 (36 to 67)	33 (18 to 49)
IIIC	17 (7 to 31)	30 (20 to 39)	38 (23 to 53)	26 (11 to 43)	15 (4 to 33)
3-year OS (95% CI)					
I	86 (77 to 92)	90 (85 to 93)	87 (79 to 92)	93 (74 to 98)	94 (86 to 97)
II	73 (65 to 79)	77 (74 to 80)	77 (72 to 82)	74 (66 to 80)	79 (72 to 85)
IIIA	52 (46 to 59)	63 (58 to 68)	62 (55 to 68)	66 (53 to 76)	64 (55 to 71)
IIIB	46 (32 to 59)	43 (34 to 51)	45 (33 to 57)	43 (27 to 58)	33 (18 to 49)
IIIC	17 (7 to 31)	23 (14 to 33)	30 (15 to 46)	16 (4 to 33)	15 (4 to 33)
Hazard ratios (95% CI)					
II	2.37 (1.26 to 4.46)	2.17 (1.52 to 3.11)	1.88 (1.16 to 3.05)	2.93 (1.06 to 8.09)	2.17 (1.12 to 4.24)
IIIA	4.86 (2.68 to 8.40)	3.73 (2.61 to 5.34)	3.67 (2.29 to 5.86)	3.59 (1.26 to 10.27)	3.99 (2.08 to 7.65)
IIIB	6.79 (3.47 to 13.27)	6.99 (4.75 to 10.27)	5.33 (3.16 to 8.97)	7.80 (2.71 to 22.51)	11.96 (5.86 to 24.42)
IIIC	16.86 (8.57 to 33.17)	13.70 (9.14 to 20.54)	9.90 (5.64 to 17.38)	15.85 (5.44 to 46.16)	23.10 (10.83 to 49.28)

Abbreviations: AL-ISS, AL International Staging System; NR, not reached; OS, overall survival.

### Role of dFLC

On multivariable analysis, dFLC ≥ 180 mg/L was not predictive of outcome in the presence of AL-ISS biomarkers in derivation and validation cohorts (Table 2), nor was it independently predictive as a continuous variable (*P* = .92, *P* = .99) or at the higher threshold of 500 mg/L (*P* = .13, *P* = .21) in both data sets, respectively (data not shown).

### Model Characteristics for Validation Cohort

**FIG 2.**
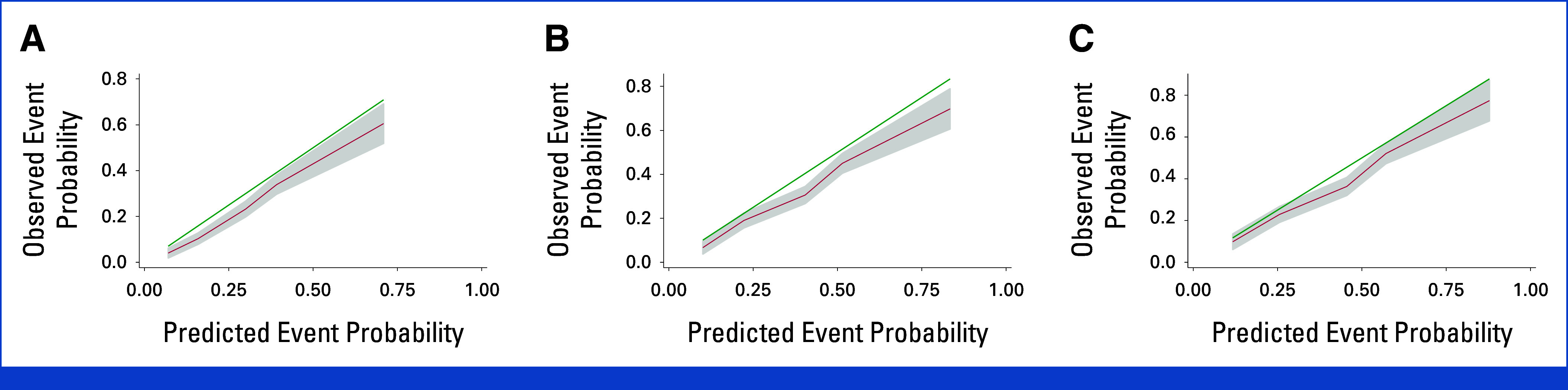
Calibration plot for (A) 12 months, (B) 24 months, and (C) 36 months. Green line shows perfect calibration and red smoothed pseudovalues with 95% CI over time.

Calibration plots are shown in Figure 2, outlining observed and predicted events. 1-year, 2-year, and 3-year calibration slopes were 1.09 (95% CI, 0.93 to 1.26, *P* = .28), 0.97 (95% CI, 0.82 to 1.11, *P* = .64), and 0.97 (95% CI, 0.82 to 1.11, *P* = .66), respectively. There was miscalibration in the large as the absolute risk estimates were reduced in validation compared with derivation cohorts as seen in the calibration curve with overestimated risk compared with historic derivation across all time points (1-year intercept –0.38 [95% CI, –0.51 to –0.21]; 2-year –0.28 [95% CI, –0.40 to –0.16]; 3-year –0.19 [95% CI, –0.29 to –0.100], *P* < .001), likely because of improved outcomes over time. Harrell C index was 0.69 (95% CI, 0.67 to 0.71), Royston D 1.19, and explained variation (R^2^_D_) was 0.25 (95% CI, 0.19 to 0.31). IIIC exhibited consistently much better DOR, higher specificity, and lower sensitivity than previous model high-risk categories (European IIIb and Mayo IV) at all time points (Data Supplement, Table S1). One-year DOR, specificity, sensitivity for AL-ISS IIIC, European IIIb, and Mayo IV were: DOR, 9.11, 2.88. 3.59, specificity: 97.8% (95% CI, 96.8 to 98.5), 92.7% (95% CI, 91.2 to 94.0), 76.1% (95% CI, 73.6 to 78.4), and specificity: 17.0% (95% CI, 13.2 to 21.6), 18.5% (95% CI, 14.6 to 23.2), 53.0% (95% CI, 47.3 to 58.7), respectively.

### Stage IIIC Versus IIIB

When comparing IIIB versus IIIC in European/NAC validation data sets, stage IIIC, by definition, had poorer LS (–6.8 *v* –11.9%, *P* < .001), lower LV ejection fraction (41 *v* 55%, *P* = <.001), greater LV wall thickness (16 *v* 15 mm, *P* = .02), and lower supine systolic blood pressure (103 *v* 115 mm Hg, *P* < .001) with nonsignificantly higher NT-proBNP (15,317 *v* 14,476 ng/L, *P* = .56) and hs-TnT (137 *v* 124 ng/L, *P* = .19). Patients with stage IIIC had less frequent renal involvement (37 *v* 64%, *P* = .001) and lower proteinuria (0.4 *v* 1.0 g/24 h, *P* = .008; Data Supplement, Table S4). Stage IIIC discriminated the poorest outcome despite a greater proportion treated with first-line daratumumab (59% *v* 40%, *P* = .008). One-year OS for IIIC and IIIB was 44% (95% CI, 32 to 55) and 69% (95% CI, 60 to 76), respectively.

### Outcomes Across by Treatment Subgroups (European/NAC)

Across time periods (2015-2019, 2020-2024, and chronological tertiles), outcomes have improved overall (Data Supplement, Tables S5 and S6, and Figs S2 and S3). Subgroup analysis of those treated with first-line daratumumab-containing regimens (n = 592) showed improved survival across all stages for those treated with daratumumab, including those with the poorest risk (IIIC) (Data Supplement, Table S7 and Fig S4). The median follow-up for those daratumumab treated was 17 months (IQR, 11-27) compared with 50 months (IQR, 34-62) in those untreated, but the median OS was >17 months for I to IIIB. In the daratumumab-treated cohort, stage IIIC 6- and 12-month survival was 61% (95% CI, 45 to 74) and 53% (95% CI, 37 to 67%), compared with 79% (95% CI, 66 to 88) and 68% (95% CI, 54 to 78), respectively, for IIIB. A greater proportion of daratumumab-treated patients had poor risk (8% *v* 5% IIIC, *P* = .006), with greater cardiac involvement (74% *v* 68%, *P* = .008), poorer LS (–14.3 *v* –15%, *P* = .01), lower supine systolic blood pressure (118 *v* 120 mm Hg, *P* = .007), lower proteinuria (1.2 *v* 2.3 g, *P* = .001), and were younger (65 *v* 68 years, *P* < .001). Despite these differences, AL-ISS remained prognostic and discriminatory for outcome. There were no differences according to light chain isotype (Data Supplement, Fig S5).

## DISCUSSION

We define and externally validate a new staging system for AL amyloidosis, termed AL-ISS, in the largest international cohort of patients treated in the modern era (Table 4). This allows risk stratification by two cardiac biomarkers (NT-proBNP and hs-TnT) and a functional cardiac marker (LS). Stage IIIC comprises 5% of patients. This ultra-poor prognostic group has a survival of 4-7 months in a contemporary era. Current approaches are inadequate, and these patients require novel therapy. Conversely, over two-thirds have mild-moderate cardiac involvement (I to IIIA), have good long-term survival (3-year OS >50%), and even those with IIIB (approximately 10%) have a median OS > 24 months.

**TABLE 4.** Outcomes Across the Entire Data Set (n = 2,493)

Stage	AL-ISS	1-yr OS (95% CI)	2-yr OS (95% CI)	3-yr OS (95% CI)
I	Both biomarkers (NT-proBNP and hs-TnT) below threshold	96 (94 to 98)	93 (90 to 95)	89 (85 to 92)
II	NT-proBNP ≥ 332 ng/L or hs-TnT ≥ 50 ng/L	89 (87 to 91)	81 (78 to 83)	76 (73 to 79)
IIIA	NT-proBNP 332-8,500 ng/L and hs-TnT ≥ 50 ng/L	75 (72 to 78)	66 (63 to 70)	60 (56 to 63)
IIIB	NT-proBNP ≥ 8,500 ng/L and hs-TnT ≥ 50 ng/L and LS < –9%	62 (56 to 69)	50 (43 to 57)	44 (37 to 51)
IIIC	NT-proBNP ≥ 8,500 ng/L and hs-TnT ≥ 50 ng/L and LS ≥ –9%	34 (26 to 43)	26 (18 to 34)	21 (14 to 29)

Abbreviations: AL-ISS, AL International Staging System; hs-TnT, high-sensitivity troponin T; LS, longitudinal strain; OS, overall survival.

AL-ISS was derived from multiple countries, supporting its utility within clinical practice and can form a new basis for the design of prospective clinical trials. Crucially, this was valid despite differences in echocardiographic software across centers, a key potential challenge in using LS across centers. Our data confirm that LS provides functional information beyond blood-based biomarkers (NT-proBNP and hs-TnT) and is most prognostic in those with advanced cardiac involvement.

European centers had a larger proportion of cardiac involvement, associated biomarkers, and subsequent higher proportion of poor risk disease. These baseline differences in populations are likely reflective of health care access variation across countries or differences in referral patterns. Despite this, AL-ISS was independently predictive among in all cohorts and time periods and exhibited good discrimination and calibration. Other studies have identified potential variables to predict survival including systolic blood pressure^3^ or dFLC at a higher threshold of 500 mg/L,^18^ but were not independently prognostic in our study,^7^ while myocardial edema by cardiac magnetic resonance^19,20^ or ^18^F-florbetapir/florbetaben positron emission tomography/computed tomography uptake^21,22^ are not univerally available. The dFLC may be an important predictor for long-term survival,^23^ but organ function dictates outcome in the early and intermediate phase of disease.

Accurate baseline risk stratification of the disease is critical for treatment selection and clinical trial design. The staging systems developed by the Mayo group^5,24^ have resolutely underpinned AL staging over the last 2 decades with a modification in 2013 by the European group to define, a then ultra-poor-risk group: stage IIIb (Data Supplement, Table S8). Outcomes in both these poor-risk categories have substantially improved with Mayo stage IV less prognostic in a bortezomib-treated population^6^ and, in retrospective studies, significantly improved outcomes of stage IIIb in daratumumab-treated patients.^10-12^ AL-ISS builds on these well-established staging systems.

The ANDROMEDA trial, which led to daratumumab approval, excluded those with European IIIb.^9^ Our data show improvements in AL amyloidosis. The absolute risks per stage have reduced over time (calibration in the large). All stages derived benefit when daratumumab when instituted, including IIIC, supporting its widespread use. Those with traditional European modified IIIb had an OS of 20 months and therefore should not preclude entry into clinical trials.

Stage IIIC represents those with the poorest outcomes. It is highly specific (more so than European IIIb and Mayo IV) but not sensitive and so identifies the unmet need with current management, although does not capture all overall events. Achievement of long-term remissions is challenging because of end-organ dysfunction at diagnosis which is difficult to surmount. Even in the daratumumab era, those with stage IIIC had a 1-year OS of around 50%. Stage IIIC was enriched with true cardiac dysfunction and less renal involvement as seen in IIIB. A limitation of the previous staging without LS is that these are influenced by renal function and so may miscategorize high-risk patients in the absence of significant cardiac involvement, suggested by the elevated cardiac biomarkers. The next frontier in disease modification is targeting the burden of amyloid present at diagnosis. Enhancing fibril clearance and enabling early organ responses (within 6-12 months) are likely required to address those with stage IIIC. Antifibril antibody studies are underway, and such a novel strategy is required. Additionally, in the current standard of care, there should be early consideration in appropriate stage IIIC patients for heart transplantation.

We acknowledge the potential for representation bias as healthcare access varied by country, with a risk of underrepresenting the poorest-risk patients in those unable to travel distances to referral centers, which may explained the varying proportion of stages at different centers. Similarly, access to daratumumab varied, introducing heterogeneity in outcome. The measurement of LS requires standardized equipment to reduce variability in measurements, with an initial learning curve and intervendor variability,^25^ cited as limitations of LS, and the exact optimal thresholds may vary across platforms. Our data set, however, supports its prognostic value internationally despite heterogeneous platforms. Stroke volume was not collected, and echocardiographic prognostic parameter studies are ongoing. Increased follow-up in the daratumumab era will be informative for determining long-term outcome.

In conclusion, as therapies have improved, it is essential to validate staging systems to stratify risk. AL-ISS has been externally validated internationally and robustly discriminates survival and identifies a new poor-risk group and is a benchmark for future clinical trials. These patients should be the focus of novel approaches.

## References

[1] Manwani R Cohen O Sharpley F et al A prospective observational study of 915 patients with systemic AL amyloidosis treated with upfront bortezomib Blood 134 2271 2280 2019 31578202 10.1182/blood.2019000834

[2] Godara A Toskic D Albanese J et al Involved free light chains <10 mg/L with treatment predict better outcomes in systemic light-chain amyloidosis Am J Hematol 96 E20 E23 2021 33068021 10.1002/ajh.26025 PMC8436337

[3] Wechalekar AD Schonland SO Kastritis E et al A European collaborative study of treatment outcomes in 346 patients with cardiac stage III AL amyloidosis Blood 121 3420 3427 2013 23479568 10.1182/blood-2012-12-473066

[4] Palladini G Sachchithanantham S Milani P et al A European collaborative study of cyclophosphamide, bortezomib, and dexamethasone in upfront treatment of systemic AL amyloidosis Blood 126 612 615 2015 25987656 10.1182/blood-2015-01-620302

[5] Kumar S Dispenzieri A Lacy MQ et al Revised prognostic staging system for light chain amyloidosis incorporating cardiac biomarkers and serum free light chain measurements J Clin Oncol 30 989 995 2012 22331953 10.1200/JCO.2011.38.5724 PMC3675680

[6] Khwaja J Ravichandran S Bomsztyk J et al Limited utility of Mayo 2012 cardiac staging system for risk stratification of patients with advanced cardiac AL amyloidosis—Analysis of a uniformly treated cohort of 1,275 patients Haematologica 109 1598 1602 2024 38205538 10.3324/haematol.2023.284348 PMC11063834

[7] Khwaja J Ravichandran S Bomsztyk J et al Refining prognostication in systemic AL amyloidosis: Limited value of dFLC Amyloid 31 353 355 2024 39311543 10.1080/13506129.2024.2406845

[8] Saunders B Theodorakakou F Fotiou DV et al Predictive value of free light chain burden in patients with AL amyloidosis treated with bortezomib-based regimens Blood Adv 9 3771 3779 2025 40305663 10.1182/bloodadvances.2024015528 PMC12309605

[9] Kastritis E Palladini G Minnema MC et al Daratumumab-based treatment for immunoglobulin light-chain amyloidosis N Engl J Med 385 46 58 2021 34192431 10.1056/NEJMoa2028631

[10] Oubari S Hegenbart U Schoder R et al Daratumumab in first-line treatment of patients with light chain amyloidosis and Mayo stage IIIb improves treatment response and overall survival Haematologica 109 220 230 2024 37439344 10.3324/haematol.2023.283325 PMC10772504

[11] Chakraborty R Rosenbaum C Kaur G et al First report of outcomes in patients with stage IIIb AL amyloidosis treated with Dara-VCD front-line therapy Br J Haematol 201 913 916 2023 36896578 10.1111/bjh.18733

[12] Khwaja J Ravichandran S Cohen O et al Outcomes of daratumumab-bortezomib-thalidomide-dexamethasone in treatment-naive systemic AL amyloidosis Br J Haematol 206 1141 1148 2025 39980430 10.1111/bjh.20021 PMC11985371

[13] Cohen OC Ismael A Pawarova B et al Longitudinal strain is an independent predictor of survival and response to therapy in patients with systemic AL amyloidosis Eur Heart J 43 333 341 2022 34472567 10.1093/eurheartj/ehab507

[14] Palladini G Schönland SO Sanchorawala V et al Clarification on the definition of complete haematologic response in light-chain (AL) amyloidosis Amyloid 28 1 2 2021 33410355 10.1080/13506129.2020.1868810

[15] Badano LP Kolias TJ Muraru D et al Standardization of left atrial, right ventricular, and right atrial deformation imaging using two-dimensional speckle tracking echocardiography: A consensus document of the EACVI/ASE/Industry Task Force to standardize deformation imaging Eur Heart J Cardiovasc Imaging 19 591 600 2018 29596561 10.1093/ehjci/jey042

[16] Royston P Explained variation for survival models Stata J 6 83 96 2006

[17] Royston P Tools for checking calibration of a Cox model in external validation: Approach based on individual event probabilities Stata J 14 738 755 2014

[18] Basset M Milani P Foli A et al Early cardiac response is possible in stage IIIb cardiac AL amyloidosis and is associated with prolonged survival Blood 140 1964 1971 2022 35772003 10.1182/blood.2022016348

[19] Porcari A Masi A Martinez-Naharro A et al Redefining cardiac involvement and targets of treatment in systemic immunoglobulin AL amyloidosis JAMA Cardiol 9 982 989 2024 39167388 10.1001/jamacardio.2024.2555 PMC11339700

[20] Kotecha T Martinez-Naharro A Treibel TA et al Myocardial edema and prognosis in amyloidosis J Am Coll Cardiol 71 2919 2931 2018 29929616 10.1016/j.jacc.2018.03.536

[21] Clerc OF Datar Y Cuddy SAM et al Prognostic value of left ventricular (18)F-Florbetapir uptake in systemic light-chain amyloidosis JACC Cardiovasc Imaging 17 911 922 2024 39001731 10.1016/j.jcmg.2024.05.002 PMC11975406

[22] Vergaro G Aimo A Genovesi D et al Estimated total amyloid burden from 18F-florbetaben positron emission tomography predicts all-cause mortality in light-chain cardiac amyloidosis Eur Heart J Cardiovasc Imaging 26 500 508 2025 39711173 10.1093/ehjci/jeae332

[23] Muchtar E Therneau TM Larson DR et al Comparative analysis of staging systems in AL amyloidosis Leukemia 33 811 814 2019 30675009 10.1038/s41375-018-0370-z

[24] Dispenzieri A Gertz MA Kyle RA et al Serum cardiac troponins and N-terminal pro-brain natriuretic peptide: A staging system for primary systemic amyloidosis J Clin Oncol 22 3751 3757 2004 15365071 10.1200/JCO.2004.03.029

[25] Shiino K Yamada A Ischenko M et al Intervendor consistency and reproducibility of left ventricular 2D global and regional strain with two different high-end ultrasound systems Eur Heart J Cardiovasc Imaging 18 707 716 27330151 10.1093/ehjci/jew120

