# Supplementary material for: New Validated Staging System for Light Chain (AL) Amyloidosis With Stage IIIC Defining Ultra-Poor Risk: AL International Staging System
Source: J Clin Oncol. 2025 Dec 7;44(4):311–20. doi: 10.1200/JCO-25-02558 (PMC12834270; doi:10.1200/JCO-25-02558)
Supplement: Supplementary file 1 [file jco-44-311-s001.pdf]

Supplemental materials

Supplemental methods

The method of echocardiographic evaluation was conducted as per centre below:

NAC, UK: Echocardiographic evaluation was performed using a GE Vivid E9 ultrasound equipped with a 5S probe and measured offline using EchoPAC software (Version 202). The overall, basal and apical LS%, LV ejection fraction, and LV wall thickness were performed and calculated in accordance with previously published guidance [15].

Mayo, USA: GE E95 platform and analysed on the GE software (on the machine or offline analysis) (version 204).

Athens, Greece: The same operator performed all echocardiograms who was blinded to the clinical information of the patients. The echocardiography system used was the Vivid S70, GE Medical Systems, Milwaukee, WI, USA. Images were captured in video loops and then were then processed using a dedicated 2D speckle tracking software (EchoPAC PC version 204; 14 GE Medical Systems, Milwaukee, WI, USA). Three apical views were used to calculate the global longitudinal strain of the left ventricle .

Pavia, Italy: Siemens Acuson SC2000 with a dedicated workstation (Syngo) for offline measurements, available for all 4 chambers.

UMCU, Netherlands: GE Vivid E9 ultrasound equipped with a 5S probe and measured offline using EchoPAC software (Version 203). The overall, basal and apical LS%, LV ejection fraction, and LV wall thickness were performed and calculated in accordance with previously published guidance.

Switzerland: GE Vivid E9 ultrasound equipped with a 5S probe and measured online using EchoPAC software.

Supplemental results

The number of patients included in the validation cohort by centre

Centre	Included in study	Total patients at centre
NAC	1088	1397
Mayo	458	941
Greece	163	337
Italy	118	1182
Netherlands	59	119
Switzerland	34	80

Supplemental table 1. AL-ISS model performance

Performance	Derivation 2015-2019	Total validation cohort	NAC validation 2020-2024	European 2015-2024	Mayo 2015-2024	Daratumumab treated NAC/European
Royston and Sauerbrei's D (R^2_D) (95% CI)		0.25 (0.19-0.31)	0.21 (0.14-0.29)	0.22 (0.12-0.34)	0.36 (0.24-0.49)	0.41 (0.26-0.55)
**Discrimination**						
Harrell's C (95% CI)		0.69 (0.67-0.71)	0.67 (0.64-0.70)	0.67 (0.62-0.72)	0.73 (0.69-0.77)	0.73 (0.68-0.78)
Calibration slope (95% CI)						
1 year		1.09 (0.93-1.26) p=0.28	0.93 (0.69-1.16) p=0.54	1.12 (0.80-1.45) p=0.46	1.74 (1.19-2.28) p=0.008	1.97 (0.75-1.39) p=0.67
2 year		0.97 (0.82-1.11) p=0.64	0.84 (0.64-1.04) p=0.12	0.94 (-0.65-1.23) p=0.69	1.41 (1.02-1.80) p=0.04	0.95 (0.65-1.25) p=0.75
3 year		0.97 (0.82-1.11), p=0.66	0.85 (0.63-1.07) p=0.18	1.01 (0.70-1.32) p=0.96	1.25 (0.91-1.59) p=0.14	1.17 (0.57-1.77) p=0.58
Calibration in the large (95% CI)						
1 year		-0.28 (-0.39- -0.17) p<0.001	-0.52 (-0.81- -0.22) p<0.001	-0.18 (-0.42-0.05) p=0.12	-0.01 (-0.21- 0.18) p=0.90	-0.64 (-0.89 - -0.40) p<0.001
2 year		-0.25 (-0.35- -0.16) p<0.001	-0.41 (-0.62- -0.20) p<0.001	-0.20 (-0.41-0.01) p=0.06	-0.15 (-0.33-0.29) p=0.10	-0.74 (-0.98- -0.50) p<0.001
3 year		-0.19 (-0.29 - -0.10) p<0.001	-0.29 (-0.49- -0.08) p=0.007	-0.11 (-0.31-0.10) p=0.30	-0.09 (-0.37-0.01) p=0.04	-0.62 (-0.94- -0.30) p<0.001
1 year						
IIIC sensitivity	19.6 (13.5-27.4)	17.0 (13.2-21.6)	13.7 (9.1-20.0)	24.0 (15.2-35.5)	17.4 (10.6-27.0)	21.2 (15.5-28.2)
IIIC specificity	98.1 (96.2-99.1)	97.8 (96.8-98.5)	97.7 (96.3-98.6)	96.7 (93.6-98.4)	98.9 (97.2-99.7)	97.1 (95.5-98.1)
Diagnostic odds ratio	12.59	9.11	6.74	9.25	18.93	9.01
2 year						
IIIC sensitivity	15.5 (10.8-21.7)	14.2 (11.1-17.8)	11.4 (7.8-16.4)	20.0 (12.9-29.4)	14.7 (9.0-22.7)	18.4 (13.5-24.5)
IIIC Specificity	98.3 (96.2-99.3)	98.0 (96.9-98.8)	97.9 (96.0-98.9)	97.0 (93.3-98.8)	98.9 (96.7-99.8)	96.8 (94.6-98.2)
Diagnostic odds ratio	10.61	8.11	6.00	8.08	15.49	6.82
3 year						
IIIC sensitivity	13.4 (9.3-10.8)	13.5 (10.7-16.9)	11.0 (7.6-15.5)	19.5 (12.9-28.2)	13.4 (8.2-20.8)	18.0 (13.3-23.8)
IIIC specificity	98.4 (96.3-99.5)	97.9 (96.4-98.8)	97.3 (94.4-98.8)	97.8 (93.8-99.6)	98.6 (96.0-99.7)	96.4 (93.2-98.1)
Diagnostic odds ratio	9.52	7.28	4.45	10.77	10.90	5.88

Performance	Derivation 2015-2019	Total validation cohort	NAC validation 2020-2024	European 2015-2024	Mayo 2015-2024	Daratumumab treated NAC/European
**Comparison to previous models**						
Harrell's C (95% CI)						
Mayo 2012	0.66 (0.63-0.69)	0.66 (0.64-0.68)	0.64 (0.60-0.67)	0.64 (0.59-0.69)	0.71 (0.67-0.74)	0.69 (0.64-0.73)
European 2015	0.68 (0.65-0.71)	0.69 (0.66-0.71)	0.67 (0.63-0.70)	0.67 (0.62-0.71)	0.73 (0.70-0.76)	0.73 (0.68-0.77)
Royston and Sauerbrei's D (R^2_D) (95% CI)						
Mayo 2012	-	0.15 (0.11-0.20)	0.11 (0.06-0.18)	0.14 (0.06-0.26)	0.21 (0.11-0.32)	0.30 (0.13-0.41)
European 2015		0.23 (0.18-0.29)	0.19 (0.12-0.27)	0.20 (0.10-0.31)	0.34 (0.22-0.46)	0.40 (0.26-0.52)
1 year						
European IIIb sensitivity	35.5 (27.7-44.2)	18.5 (14.6-23.2)	29.8 (23.1-37.4)	45.3 (33.9-57.2)	38.0 (28.3-48.8)	42.9 (35.5-50.8)
European IIIb specificity	91.3 (88.2-93.7)	92.7 (91.2-94.0)	89.3 (86.8-91.3)	87.8 (83.2-91.4)	95.0 (92.1-96.9)	90.7 (88.3-92.7)
Diagnostic odds ratio	5.78	2.88	3.54	5.96	11.65	7.33
Mayo IV sensitivity	51.5 (42.7-60.2)	53.0 (47.3-58.7)	43.9 (36.2-51.9)	69.0 (55.3-80.1)	59.3 (48.5-69.4)	62.5 (50.3-73.4)
Mayo IV specificity	74.1 (69.5-78.3)	76.1 (73.6-78.4)	78.0 (74.8-80.9)	60.9 (52.8-68.4)	78.9 (74.2-82.9)	66.9 (61.4-72.0)
Diagnostic odds ratio	3.04	3.59	2.77	3.47	5.45	3.37
2 year						
European IIIb sensitivity	29.9 (23.6-37.1)	17.7 (14.4-21.6)	28.0 (22.4-34.2)	39.0 (29.6-49.3)	33.6 (25.3-43.1)	39.1 (32.5-46.2)
European IIIb Specificity	92.2 (88.9-94.7)	94.0 (92.2-95.3)	91.1 (88.1-93.5)	88.6 (83.1-92.5)	95.8 (92.5-97.7)	92.5 (89.5-94.7)
Diagnostic odds ratio	5.04	3.37	3.98	4.97	11.54	7.92
Mayo IV sensitivity	48.6 (41.1-56.2)	48.3 (43.3-53.2)	39.9 (33.6-46.6)	63.0 (50.9-73.8)	55.6 (46.1-64.8)	61.0 (49.5-71.4)
Mayo IV specificity	76.2 (71.3-80.6)	77.8 (74.9-80.6)	80.7 (76.7-84.2)	60.2 (20.7-68.9)	80.6 (75.4-84.9)	65.0 (56.3-72.8)
Diagnostic odds ratio	3.03	3.27	2.78	2.57	5.20	2.90
3 year						
European IIIb sensitivity	26.3 (20.7-32.7)	17.3 (14.1-20.9)	27.3 (22.1-33.1)	38.9 (30.0-48.6)	30.7 (23.0-39.6)	37.8 (31.5-44.6)
European IIIb specificity	92.7 (89.1-95.2)	94.4 (92.2-96.0)	91.7 (87.5-94.6)	88.5 (81.7-93.1)	95.4 (91.5-97.7)	91.6 (87.5-94.5)
Diagnostic odds ratio	4.53	3.53	4.15	4.90	9.19	6.62
Mayo IV sensitivity	46.2 (39.3-53.2)	46.2 (41.6-50.9)	38.8 (32.9-45.1)	61.9 (56.6-72.1)	50.8 (41.8-59.8)	61.6 (50.5-71.7)
Mayo IV specificity	77.6 (72.4-82.0)	78.7 (75.0-82.0)	82.3 (76.9-86.7)	61.4 (50.1-71.7)	81.2 (75.2-86.0)	57.7 (43.6-71.0)
Diagnostic odds ratio	2.97	3.17	2.95	2.58	4.46	2.19

Supplemental table 2. Performance of LS thresholds for European IIIb (derivation cohort)

	**LS ≥-9%**	**LS ≥-10%**
1 year		
Sensitivity (95% CI)	55.1 (40.3-69.1)	59.2 (44.3-72.7)
Specificity (95% CI)	78.4 (61.3-89.6)	64.9 (47.2-79.3)
Diagnostic odds ratio	4.45	2.68
2 year		
Sensitivity (95% CI)	51.8 (38.2-65.2)	57.1 (43.3-70.0)
Specificity (95% CI)	78.6 (59.0-91.7)	64.3 (44.1-81.4)
Diagnostic odds ratio	3.95	2.40
3 year		
Sensitivity (95% CI)	47.2 (24.2-70.1)	56.1 (42.4-69.0)
Specificity (95% CI)	78.3 (56.3-92.5)	60.9 (38.5-80.3)
Diagnostic odds ratio	3.23	1.99

AUC for LS prediction of 1-year survival is 0.63 in European IIIb derivation cohort; optimal LS 1-year ROC cut-off was -9.2%: sensitivity 59% (95% CI 44-73), specificity 76% (95% CI 58-88). For the entire derivation population, LS≥-9% had a high 1-year specificity for OS at 90.6% (95% CI 87.4-93.1) with low sensitivity 34.8% (95% CI 27.0-43.4). The rounded LS≥-9% threshold at 1-year was chosen as this was both clinically meaningful and had a low rate of censoring (8/573 patients in the derivation cohort, 1/87 patients with European IIIb).

Supplemental table 3. Proportion of LS ≥-9% by AL-ISS

	Proportion with LS ≥-9%			
	**Stage I**	**Stage II**	**Stage IIIA**	**Stage IIIC**
**Derivation** (n=573)	1 (1)	12 (7)	41 (18)	35 (100)
**All validation**				
NAC (n=987/1088)	1 (1)	22 (6)	53 (18)	49 (100)
European (n=374)	0 (0)	10 (5)	9 (11)	27 (100)
Mayo (n=458)	0(0)	11(6)	28 (19)	20 (100)

Supplemental table 4. Baseline characteristics of stage IIIB v IIIC (European/NAC)

	**Stage IIIB**<br>n=141	**Stage IIIC**<br>n=76	**P value**
Age (years), median (IQR)	70 (61-75)	67 (60-74)	0.19
Male<br>Female	87 (62)<br>54 (38)	51 (67)<br>25 (33)	0.40
AL isotype<br>λ type<br>κ type	 98 (70)<br>43 (31)	 55 (72)<br>21 (28)	 0.66
Bone marrow plasma cell infiltrate (%), median (IQR)	15 (10-25)	18 (10-28)	0.91
dFLC (mg/L), median (IQR)	338 (132-640)	346 (173-613)	0.65
Monoclonal protein (g/L), median (IQR)	0 (0-8)	0 (0-4)	0.23
Organ involvement, n (range)	2 (1-5)	2 (1-5)	0.48
Organ involvement<br>Cardiac<br>Renal	 137 (97)<br>89 (64)	 76 (100)<br>28 (37)	 0.14<br>0.001
NT-proBNP (ng/L), median (IQR)	14476 (10956-20864)	15317 (10671-26082)	0.54
Hs-Troponin T (ng/L), median (IQR)	124 (80-179)	137 (95-213)	0.14
Longitudinal strain (%), median (IQR)	-11.9 (-14.1 - -10.3)	-6.8 (-7.9 - -5.7)	<0.001
LV EF (%), median (IQR)	55 (49-60)	41 (35-50)	<0.001
LV septal thickness (mm), median (IQR)	15 (14-16)	16 (14-17)	0.03
Supine systolic blood pressure (mmHg), median (IQR)	114 (102-131)	105 (95-113)	<0.001
Creatinine (µmmol), median (IQR)	132 (100-248)	108 (89-139)	0.001
Proteinuria (g/24 hrs), median (IQR)	1.0 (0.3-4.2)	0.4 (0.2-1.5)	0.009
ALP (U/L), median (IQR)	110 (86-145)	136 (84-179)	0.13
Albumin (g/L), median (IQR)	36 (30-40)	39 (32-42)	0.03
First-line daratumumab-based	58 (41)	45 (59)	0.011

Supplemental table 5. Outcomes over time periods 2015-2019 v 2020-2024 (European/NAC)

	**2015-2019**	**2020-2024**
	n=668	n=1367
**Median follow up, months (IQR)**	63 (55-76)	28 (17-43)
Number		
I	97 (15)	219 (16)
II	222 (33)	564 (42)
IIIA	248 (37)	381 (28)
IIIB	60 (9)	133 (10)
IIIC	41 (6)	70 (5)
1 year OS (95% CI)		
Stage I	97 (91-99)	94 (90-97)
Stage II	87 (82-91)	90 (87-92)
Stage IIIA	72 (66-77)	79 (74-82)
Stage IIIB	62 (48-73)	68 (59-75)
Stage IIIC	24 (13-38)	45 (33-56)
2 year OS (95% CI)		
Stage I	93 (86-96)	91 (86-95)
Stage II	78 (71-83)	81 (77-84)
Stage IIIA	61 (54-67)	70 (64-74)
Stage IIIB	53 (39-65)	52 (43-61)
Stage IIIC	17 (8-30)	35 (23-47)
3 year OS (95% CI)		
Stage I	86 (77-92)	88 (82-92)
Stage II	72 (66-78)	77 (73-81)
Stage IIIA	54 (47-60)	63 (57-68)
Stage IIIB	47 (34-59)	44 (34-54)
Stage IIIC	17 (8-30)	24 (13-37)

Supplemental table 6. Outcomes by chronological tertiles (European/NAC)

	**Tertile 1 January 2015-January 2020**	**Tertile 2 January 2020-August 2022**	**Tertile 3 August 2022-December 2024**
	n=679	n=678	n=678
**Median follow up, months (IQR)**	63 (54-51)	44 (37-51)	17 (11-24)
Number			
I	102 (15)	105 (15)	110 (16)
II	228 (34)	260 (38)	298 (44)
IIIA	248 (37)	199 (29)	182 (27)
IIIB	61 (9)	72 (11)	60 (9)
IIIC	41 (6)	42 (6)	28 (4)
1 year OS (95% CI)			
Stage I	96 (90-98)	94 (88-97)	95 (89-98)
Stage II	87 (82-91)	87 (82-90)	92 (89-95)
Stage IIIA	72 (66-77)	73 (66-78)	86 (80-90)
Stage IIIB	62 (49-73)	67 (54-76)	69 (55-79)
Stage IIIC	24 (13-38)	45 (30-59)	44 (24-63)
2 year OS (95% CI)			
Stage I	92 (85-96)	91 (84-95)	-
Stage II	78 (72-83)	77 (71-81)	-
Stage IIIA	61 (54-67)	62 (54-68)	-
Stage IIIB	52 (39-64)	52 (40-63)	-
Stage IIIC	17 (8-30)	33 (20-48)	-
3 year OS (95% CI)			
Stage I	86 (77-91)	88 (80-93)	-
Stage II	72 (66-78)	72 (66-77)	-
Stage IIIA	54 (47-60)	56 (48-62)	-
Stage IIIB	46 (33-58)	44 (32-55)	-
Stage IIIC	17 (8-30)	23 (12-37)	-

Tertile 3: 1-year OS reported due to limited follow up

Supplemental table 7. Outcomes by treatment group (European/NAC)

	Non daratumumab-treated	Daratumumab-treated
	n=1443	n=592
**Median follow up, months (IQR)**	50 (34-62)	17 (11-27)
Number		
I	235 (16)	81 (14)
II	542 (38)	244 (41)
IIIA	465 (32)	164 (28)
IIIB	135 (9)	58 (10)
IIIC	66 (5)	45 (8)
Median OS, months (95% CI)		
I	NR	NR
II	NR	NR
IIIA	67 (36-NR)	NR
IIIB	26 (19-50)	NR
IIIC	5 (3-8)	18 (5-NR)
1 year OS (95% CI)		
Stage I	94 (90-96)	99 (92-100)
Stage II	87 (84-90)	93 (88-96)
Stage IIIA	73 (69-77)	86 (79-90)
Stage IIIB	65 (57-73)	68 (52-78)
Stage IIIC	27 (17-38)	53 (37-67)
2 year OS (95% CI)		
Stage I	90 (85-93)	99 (92-100)
Stage II	78 (74-81)	89 (83-93)
Stage IIIA	62 (57-66)	80 (71-86)
Stage IIIB	52 (43-60)	59 (43-72)
Stage IIIC	17 (9-27)	49 (33-64)
3 year OS (95% CI)		
Stage I	85 (80-89)	99 (92-100)
Stage II	73 (69-76)	85 (74-92)

Stage IIIA	55 (50-59)	80 (71-86)
Stage IIIB	45 (36-53)	52 (34-68)
Stage IIIC	14 (7-23)	38 (21-56)

Supplemental table 8. Staging systems in systemic AL amyloidosis

	Mayo 2004	European modified Mayo 2004	Mayo 2012	AL-ISS
**Stage I**	No biomarkers above threshold	No biomarkers above threshold	No biomarkers above threshold	NT-proBNP *and* hs-TnT below threshold
**Stage II**	NT-proBNP ≥ 332 ng/L *or* hs-Tn T≥ 50 ng/L	NT-proBNP ≥ 332 ng/L *or* hs-TnT ≥ 50 ng/L	One of: NT-proBNP ≥ 1800 ng/L, hs-TnT ≥ 40ng/L, dFLC ≥ 180mg/L	NT-proBNP ≥ 332 ng/L *or* hs-TnT ≥ 50 ng/L
**Stage III /European IIIa**	Two biomarkers above threshold	NT-proBNP 332-8500 ng/L *and* hs-TnT ≥ 50 ng/L	Two biomarkers above threshold	NT-proBNP 332-8500 ng/L *and* hs-TnT ≥ 50 ng/L
**Stage IIIb**	-	NT-proBNP ≥ 8500 ng/L *and* hs-TnT ≥ 50 ng/L	-	NT-proBNP ≥ 8500 ng/L *and* hs-TnT ≥ 50 ng/L *and* LS < -9%
**Stage IV**	-	-	Three biomarkers above threshold	-
**Stage IIIC**	-	-	-	NT-proBNP ≥8500 ng/L *and* hs-TnT ≥50 ng/L *and* LS ≥ -9%

Biomarker thresholds: hs-TnT 50 ng/L is equivalent to cTnT 0.035 μg/L or cTnI 0.1ng/L; hs-TnT 40 ng/L equivalent to cTnT 0.025 μg/L

Supplemental figure 1. Survival by LS ≥-9% by European stage (validation)

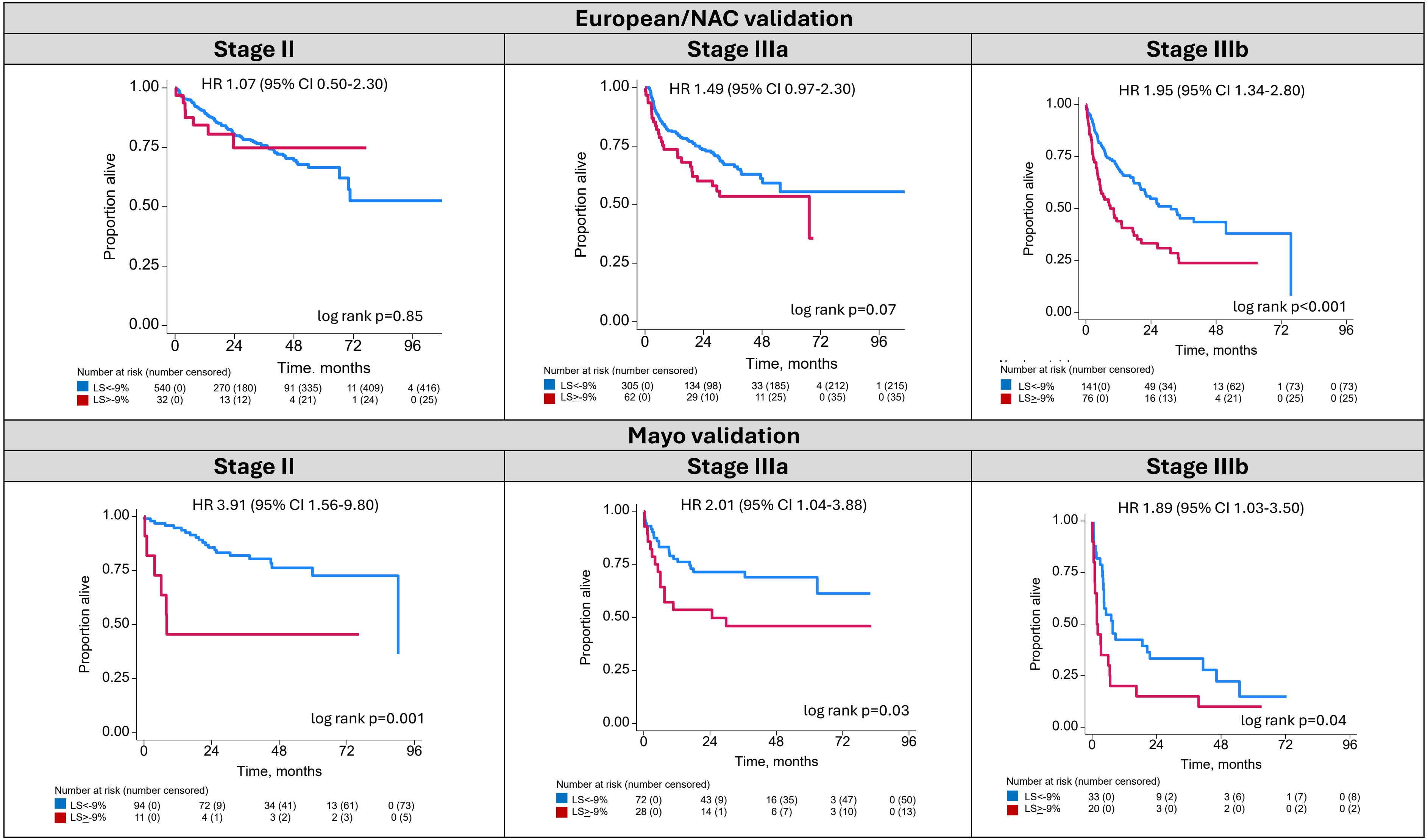

Stage I was not generated as only 1 patient

Supplemental figure 2. Outcomes over time by AL-ISS (2015-2019 v 2020-2024: European/NAC)

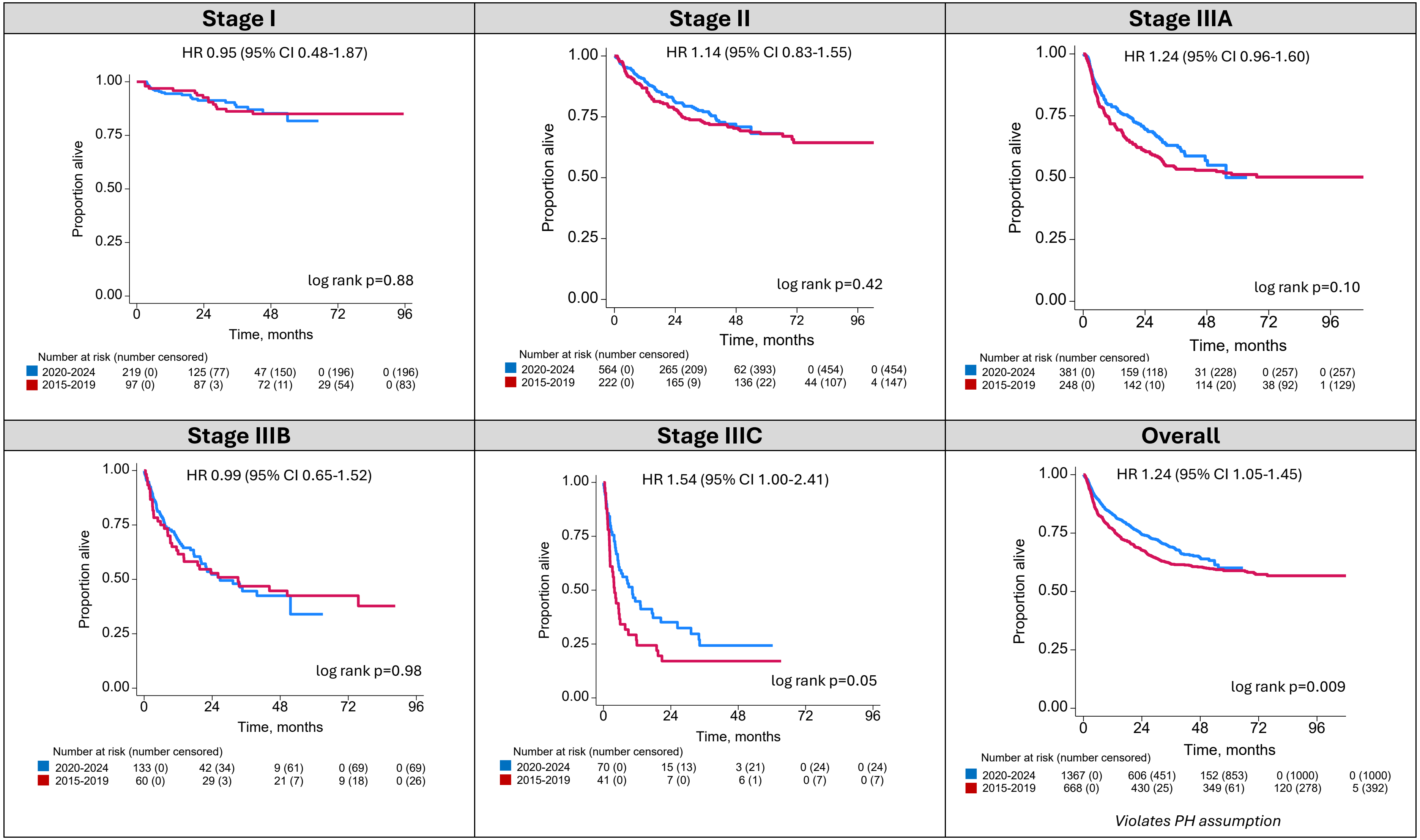

Where PH assumption was violated, the log rank test remains valid however the HR can only be taken as an average over time.

Supplemental figure 3. Outcomes over chronological tertiles by AL-ISS (European/NAC)

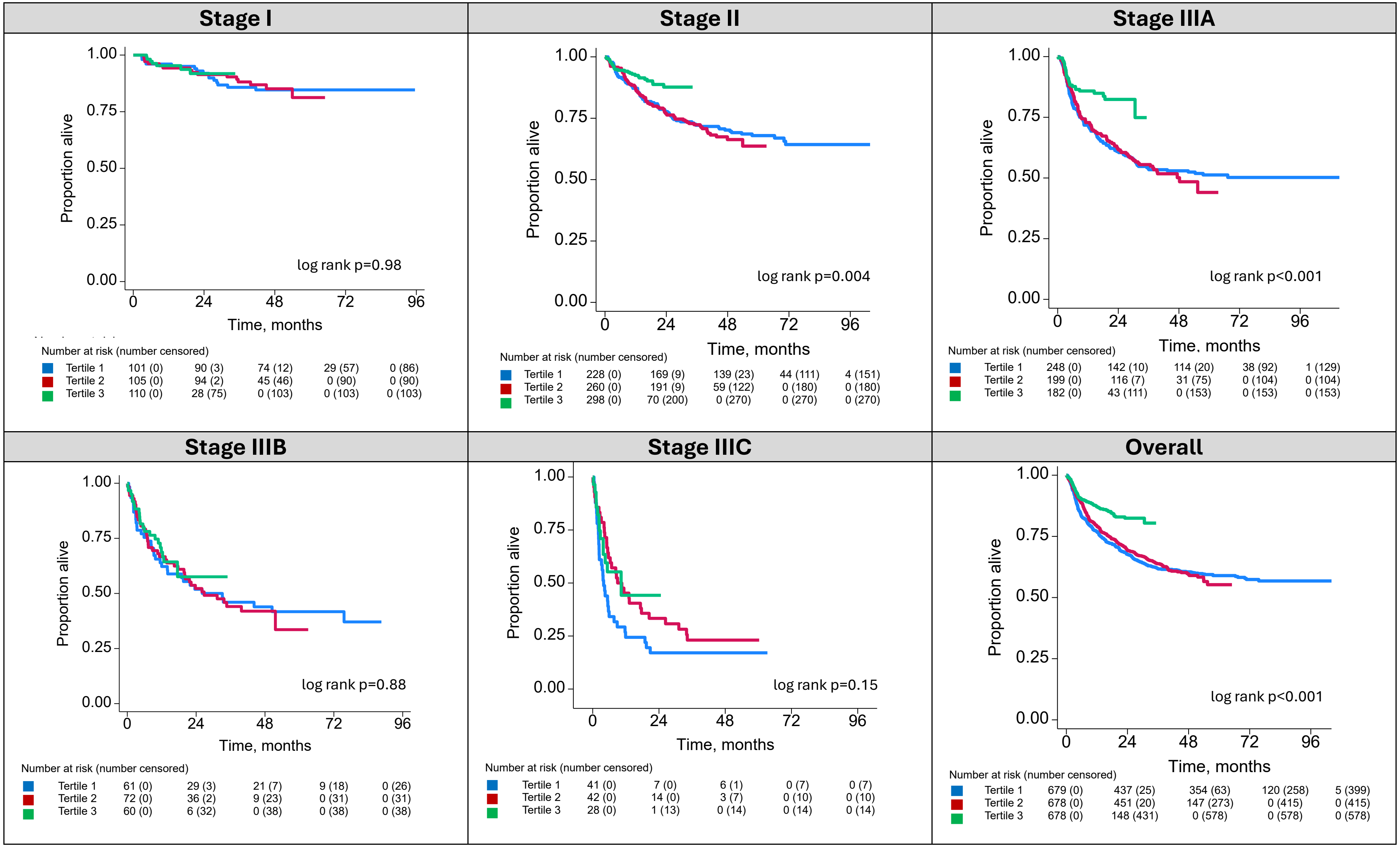

Supplemental figure 4. Outcomes of patients treated with daratumumab by AL-ISS (European/NAC)

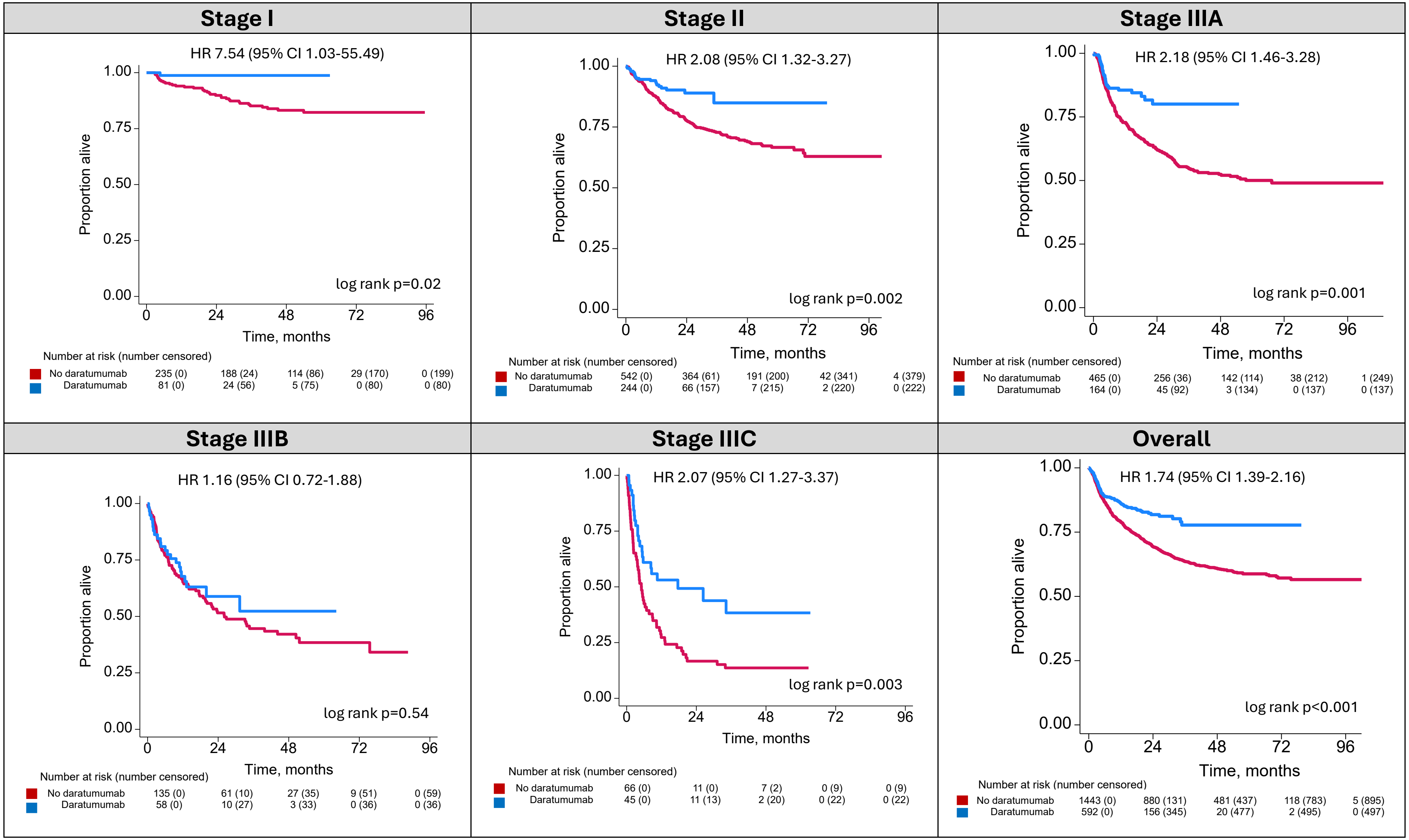

Supplemental figure 5. Outcomes by light chain isotype and AL-ISS, European staging and daratumumab-exposure (European/NAC)

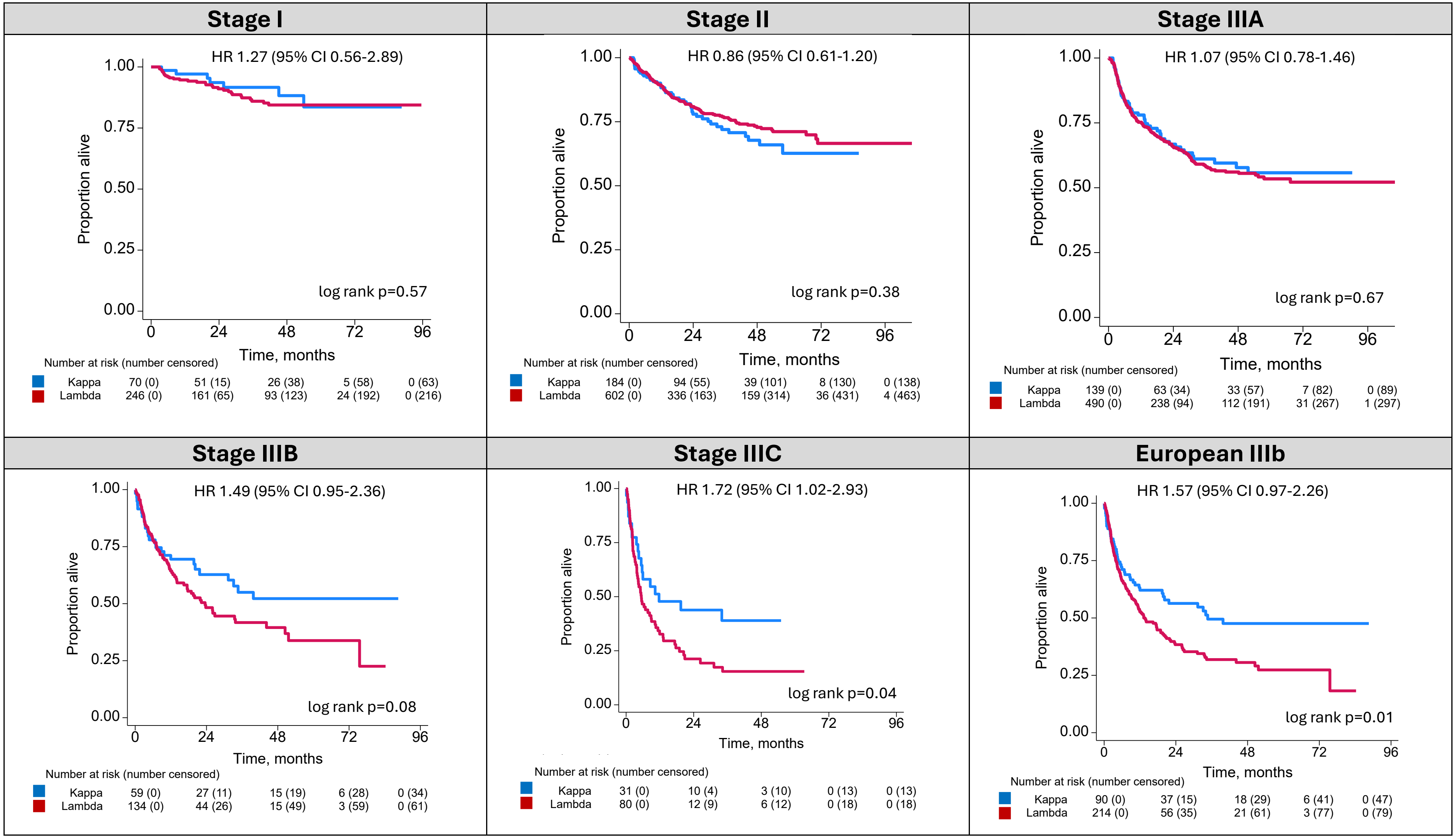

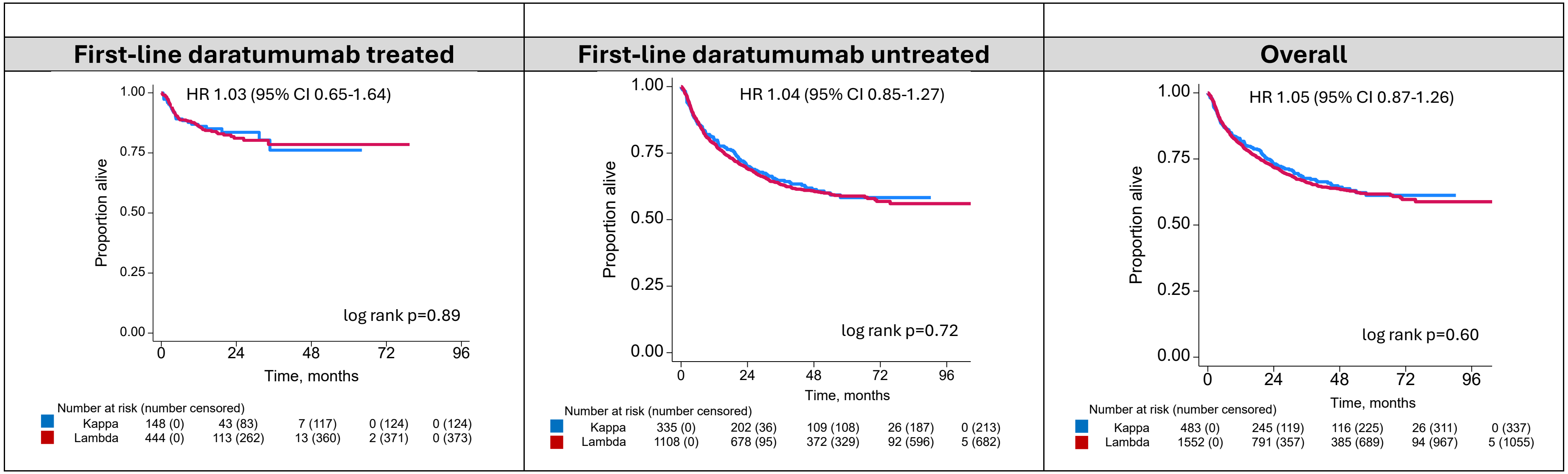
First-line daratumumab treated
HR 1.03 (95% CI 0.65-1.64)
log rank p=0.89
Proportion alive
1.00
0.75
0.50
0.25
0.00
0
24
48
72
96
Time, months
Number at risk (number censored)
Kappa 148 (0) 43 (83) 7 (117) 0 (124) 0 (124)
Lambda 444 (0) 113 (262) 13 (360) 2 (371) 0 (373)
First-line daratumumab untreated
HR 1.04 (95% CI 0.85-1.27)
log rank p=0.72
Proportion alive
1.00
0.75
0.50
0.25
0.00
0
24
48
72
96
Time, months
Number at risk (number censored)
Kappa 335 (0) 202 (36) 109 (108) 26 (187) 0 (213)
Lambda 1108 (0) 678 (95) 372 (329) 92 (596) 5 (682)
Overall
HR 1.05 (95% CI 0.87-1.26)
log rank p=0.60
Proportion alive
1.00
0.75
0.50
0.25
0.00
0
24
48
72
96
Time, months
Number at risk (number censored)
Kappa 483 (0) 245 (119) 116 (225) 26 (311) 0 (337)
Lambda 1552 (0) 791 (357) 385 (689) 94 (967) 5 (1055)
